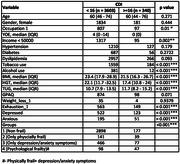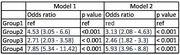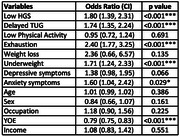# Differential Association of Frailty Types with Cognitive Functional Impairment

**DOI:** 10.1002/alz.091695

**Published:** 2025-01-09

**Authors:** Sakshi Arora, Raghav Prasad, Jonas S. Sundarakumar, Pooja Rai

**Affiliations:** ^1^ Centre for Brain Research, Indian Institute of Science, Bangalore, Karnataka India

## Abstract

**Background:**

The phenotype model of frailty, initially proposed by Fried, is primarily confined to the physical dimension. This study seeks to expand this model by integrating the psychological dimension. We hypothesize that this psychological frailty model is strongly associated with cognitive functional impairment (CFI) in an aging Indian population.

**Method:**

We used baseline cross‐sectional data (n = 3943) from the ongoing Srinivaspura Aging, Neuro Senescence, and COGnition (SANSCOG) study. Physical frailty was evaluated using a modified version of Fried’s criteria, encompassing hand grip strength (HGS), gait speed (timed up and go, TUG), physical activity levels, self‐reported exhaustion, and weight loss (self‐reported or a BMI < 18.5). Depression/ anxiety symptoms were assessed using the Generalized Depression Scale (GDS‐30) and Generalized Anxiety Disorder (GAD‐7) scale, respectively. Participants were classified into four groups – non‐frail, only physically frail, individuals with depression/anxiety symptoms (GDS>10/ GAD>10), and psychologically frail (physical frailty components + depression/anxiety). CFI was assessed using the Instrumental Activities of Daily Living (IADL‐E) for elderly (cognitive disability index, CDI >16). We performed logistic regression to test the association of frailty groups with cognitive functional impairment.

**Result:**

In this study, 8.6% exhibited CFI, 4.5% were physically frail and 3.7% were identified as psychologically frail. Adjusted logistic regression analysis indicated that risk of cognitive functional impairment was 5.9 times (95% CI: 4.0 ‐ 8.8, p<0.001) higher for psychologically frail, 3.1 times (95% CI: 2.1 ‐ 4.6, p<0.001) higher for physically frail, and 2.46 times (95% CI: 1.82 ‐ 3.3, p<0.001) higher for those with depression or anxiety symptoms, respectively. On performing logistic regression for individual components of psychological frailty, we found significant association of lower HGS (OR = 1.80, 1.39‐2.31, p<0.001), delayed TUG (OR = 1.74, 1.35‐2.24, p<0.001), exhaustion (OR = 2.40, 1.58‐2.90, p<0.001), underweight i.e., BMI <18.5 (OR = 1.51, 1.09‐2.05, p<0.05) and GAD (OR = 1.65, 1.08‐2.50, p<0.05) with CFI.

**Conclusion:**

Psychological frailty showed increased risk of cognitive functional impairment. This highlights the importance of a comprehensive frailty assessment in clinical practice. Therefore, while designing interventions, both physical as well as psychological health should be addressed to improve functional ability in aging population.